# Assessment of Depression and Anxiety in Patients with Chronic Kidney Disease and after Kidney Transplantation—A Comparative Analysis

**DOI:** 10.3390/ijerph181910517

**Published:** 2021-10-07

**Authors:** Wioletta Dziubek, Weronika Pawlaczyk, Lukasz Rogowski, Malgorzata Stefanska, Tomasz Golebiowski, Oktawia Mazanowska, Magdalena Krajewska, Mariusz Kusztal, Joanna Kowalska

**Affiliations:** 1 Faculty of Physiotherapy, Wroclaw University of Health and Sport Sciences, 35 Paderewskiego Street, 51-612 Wrocław, Poland; wioletta.dziubek@awf.wroc.pl (W.D.); malgorzata.stefanska@awf.wroc.pl (M.S.); joanna.kowalska@awf.wroc.pl (J.K.); 2 Lower Silesian Oncology Center, Department of Physiotherapy, pl. Hirszfelda 12, 53-413 Wroclaw, Poland; 3 Faculty of Health and Physical Culture Sciences, The Witelon State University of Applied Sciences in Legnica, 5A Sejmowa Street, 59-220 Legnica, Poland; lukasz.rogowski@gsuite.pwsz.legnica.edu.pl; 4 Department of Nephrology and Transplantation Medicine, Faculty of Medicine, Wroclaw Medical University, Borowska 213, 50-556 Wrocław, Poland; tomasz.golebiowski@umed.wroc.pl (T.G.); oktawia.mazanowska@umed.wroc.pl (O.M.); magdalena.krajewska@umed.wroc.pl (M.K.); mariusz.kusztal@umed.wroc.pl (M.K.)

**Keywords:** depressive disorders, anxiety, satisfaction with life, handgrip strength, kidney transplantation, chronic kidney disease

## Abstract

Depression and anxiety are common among chronic kidney disease (CKD) patients but are rarely diagnosed and treated. Furthermore, the fraction of patients with depression is greater among hemodialyzed patients. The aim of the study was to assess the prevalence of depression symptoms, anxiety and assess the level of life satisfaction in three groups of patients based on the stage of CKD. The study group consisted of 283 patients—130 females and 153 males, mean aged was 54.7 (±15.3) with stage III–V chronic kidney disease and after kidney transplantation. The Beck Depression Inventory (BDI), the Satisfaction with Life Scale (SWLS), and the State-Trait Anxiety Inventory (STAI) were used. The highest percentage of patients with depressive symptoms was recorded in the group of dialysis patients with stage V CKD. The lowest percentage of patients with high satisfaction with life was noted in the pre-dialysis group. There was a significant relationship between BDI and STAI, SWLS in all groups while a significant relationship between BDI and handgrip strength was observed in dialysis and kidney transplantation patients. Anxiety as a trait was found to be the factor most significantly associated with depressive symptoms in each of the three patient groups. Screen testing and monitoring of the emotional state of patients with CKD are needed, regardless of the stage of the disease and treatment, including patients after kidney transplantation.

## 1. Introduction

Chronic kidney disease (CKD) is listed among the lifestyle diseases of the 21st century. Chronic kidney disease is a multisymptomatic disease syndrome that develops due to a decrease in the number of active nephrons destroyed in the renal. It is estimated that CKD affects over 4 million people in Poland [1]. This syndrome affects not only physical but also psychological functioning.

Depression and anxiety are common among CKD patients but are rarely diagnosed and treated. Psychiatric disorders that occur in this group of patients also include dementia, psychosis and sleep disturbances [2,3]. Depressive disorders are mainly observed in patients with end-stage renal disease (ESRD) [4]; however, they can occur at any stage of CKD. Furthermore, the fraction of patients with depression is greater among hemodialyzed patients [5].

Patients with stage V disease require renal replacement therapy, which is associated with significant changes in the patient’s lifestyle, such as limitations to occupational activity and engagement in previously assumed social roles, and the need to follow strict therapeutic (systematic dialysis therapy) and dietary regiment [6]. Studies by Makara-Studzińska et al. [3] revealed a high prevalence of depressive disorders among patients with ESRD who are undergoing dialysis, in which 56.35% and 22.29% of patients had mild and moderate disorders, respectively. In a meta-analysis by Palmer et al., the prevalence of depressive disorders in patients with stages I–V of CKD was 21.4–26.5%, in patients with ESRD it was 22.8–39.3%, while in kidney transplant patients it was 25.7% and 26.6% [7]. Aggarwal et al. found that the prevalence of anxiety and depression were 71% and 69% respectively. As the CKD stage advanced, the prevalence, as well as the severity of these parameters increased [2]. 

The somatic symptoms associated with kidney disease and its treatment may mask depressive symptoms, often delaying a proper diagnosis. The diagnosis of depression and the introduction of proper therapeutic management can improve the patient’s mental state and quality of life and it may also have a positive impact on their long-term survival [8].

There are a lot of factors associated with depression and anxiety among patients with CKD, such as employment status, unemployment, low income, urban residence, female gender, older age, education level, duration of illness, and duration of hemodialysis, stage of CKD and presence of comorbidities [2,9]. According to other authors also low handgrip strength (GS) has been associated with an increase in depressive symptoms, independent of age group or gender [10,11,12].

Also, life satisfaction and well-being are notable lower in the population of hemodialysis patients compared to the general population [13]. After transplantation patients report higher life satisfaction, mostly due to the perception of greater control of health and living a normal active life with satisfying emotional relationships [14]. 

Knowing the factors related to depression and anxiety in CKD patients can help to identify high-risk persons in whom modifying these predictors can help in providing an active and healthy life or holistic treatment and rehabilitation. 

Unfortunately, very few publications look at the analysis and comparison of patients’ emotional states at different stages of CKD (pre-dialysis, dialysis patients) and patients who had undergone kidney transplantation.

Therefore the aim of the study was to assess the prevalence of depression symptoms, anxiety and assess life satisfaction in three groups of patients based on the stage of CKD and a comparative analysis of emotional status in these groups of patients. Additionally, we try to answer the question of what factors (including handgrip strength) are associated with depression symptoms in patients with CKD and in renal transplant recipients?

## 2. Materials and Methods

### 2.1. Participants

This observational study was conducted at the University Clinical Hospital in Wroclaw in 2018–2019. The study group consisted of 283 patients—130 female and 153 male, aged mean 54.7 (±15.3) with stage III–V chronic kidney disease and after kidney transplantation. Exclusion criteria were established: no consent to participate in the study, the presence of serious mental disorders (e.g., consciousness disorders or mental disorders) in the medical records or at the time of the study and current antidepressant treatment.

The study group of patients was divided into three subgroups based on the stage of CKD:

- Group 1, 87 dialysis patients with stage V CKD;
- Group 2, 94 pre-dialysis patients with stage III or IV CKD;
- Group 3, 102 patients who had undergone kidney transplantation.

The three groups of patients did not differ significantly with respect to their height, weight and BMI or gender, education and marital status. However, there was a significant difference in the place of residence. Moreover, there was a statistically significant difference in age and number of comorbidities. Patients in Group 2 were significantly younger compared to patients in Group 1 and had statistically fewer comorbidities compared to Group 3. 

Detailed patient characteristics are presented in Table 1.

### 2.2. Procedure

All patients who consented to participate in the study completed a self-administered questionnaire using pencil and paper. This took place during a doctor’s follow-up visit (pre-dialysis patients with stage III or IV CKD and patients who had undergone kidney transplantation) and before dialysis (dialysis patients with stage V CKD). The examination time was not limited and it depended on the individual characteristics of the respondent (on average about 40 min). All respondents were qualified for the program by a nephrologist.

All participants were informed about the purpose of the study and the possibility to withdraw at any stage. The study was carried out without any intervention or experiment structure, with the participants’ consent and under the ethical and legal supervision of the Bioethics Committee of the University School of Physical Education in Wroclaw, Poland (reference no 26/2017). The study was conducted in accordance with the Helsinki Declaration.

### 2.3. Measurement Tools

The study group was assessed using the following measurement scales: The Beck Depression Inventory (BDI), the Satisfaction With Life Scale (SWLS), and the State-Trait Anxiety Inventory (STAI). Additionally, a patient’s medical file was completed, containing sociodemographic (age, gender, education, marital status, place of residence) and clinical data, collected on the basis of an interview and their previous medical records. 

The BDI is a screening scale used to assess depressive symptoms in adults and adolescents. The scale consists of 21 questions (including two parts: emotional and somatic) containing four ranked descriptions of the severity of depressive symptoms, evaluated in a perspective of the past two weeks (0—no symptoms; 3—maximum symptom severity). The score of 0–11 points indicates no depression, 12–19—mild depression, 20–25—moderate depression and 26–63—severe depression. The BDI has very high internal consistency—Cronbach’s alpha for the entire standardization sample is 0.93 and for patients suffering from depression 0.95 [15].

The SWLS is a tool for measuring an individual’s subjective feeling of satisfaction with life, used to study adults. The respondent indicates the extent to which they agree with each of the five statements by selecting one of seven responses (1—Totally Disagree; 7—Totally Agree). The higher the score, the greater the sense of satisfaction with life. The scores of 5–17 indicate low satisfaction, 18–23 indicate average satisfaction and 24–35 represent high satisfaction with life. The psychometric properties of the Polish version are satisfactory and similar to the original (Cronbach’s alpha is 0.81) [16]. 

The STAI is used to diagnose the severity of situational anxiety (anxiety as a state, STAI X-1) and anxiety considered as a personality trait (anxiety as a trait, STAI X-2). The STAI X-1 consists of 20 responses. The respondents select one of four responses that describe how they feel at that moment (1—definitely no; 4—definitely yes). STAI X-2 consists of 20 responses where the respondents select one of four answers that describes how they generally feel (1—almost never; 4—almost always). The maximum score is 80 points. The obtained score indicates the level of anxiety, where the higher the score, the higher the level of anxiety. The threshold for dividing patients into subgroups with low and high levels of anxiety for the STAI X-1 was a score of 44, and for the STAI X-2, a score of 46 was used. The psychometric properties of the Polish version are similar to the original (Cronbach’s alpha is 0.89 for STAI X-1 and 0.83 for STAI X-2) [17]. 

A hydraulic dynamometer (Jamar) was used to measure the handgrip strength. The examination was carried out in a sitting position at the table. The examined limb was bent at the elbow joint to an angle of 90 degrees, three attempts of alternating the left and right hand were performed. The score was the mean score of each of the three trials [18]. The Jamar is a highly reliable (ICC = 0.98) assessment tool for measuring maximal grip strength [19].

### 2.4. Statistical Analysis

The Shapiro–Wilk test was used to examine the distribution of all parameters measured on the quantitative scale. In the majority of cases, the normality of distribution has not been confirmed. In order to describe the results, a median and inter-quartile range (IQR) were used for both quantitative and ordinal scale variables. The Kruskal–Wallis, one-way ANOVA and Mann–Whitney U test was used to assess the significance of differences between groups.

Multiple comparisons of the mean ranks package were used as a post hoc test, where the analysis of variance revealed statistical significance. The χ^2^ test was used to verify the significance of differences between groups in terms of their selected sociological traits. Spearman’s correlation analysis for selected pairs was also used. The values r < 0.2 were considered as no association, r = |0.2–0.4|weak correlation, r = |0.4–0.6|moderate correlation, r = |0.6–0.8|strong correlation. The significance level was assumed at *p* < 0.05. Multivariate regression was performed to find associations between the prevalence of depression and other variables.

Additionally, to determine the quantity of the effect of differences between examined groups, a corrected Cohen’s d test was used. The effect size of interaction was calculated by Eta squared (η^2^) and then transformed to Cohen’s d value [20]. The values of Cohen’s d test ≥ 0.8 indicated great strength of the observed effect. Multiple Regression Effect Size References were computed using Cohen’s f-square test [21]. Cramer’s V coefficient was used to calculate the effect size of χ^2^ test with more than one degree of freedom.

All calculations were made using the Statistica 13.1 and statistical calculators http://www.psychometrica.de/effect_size (access date 30 August 2021) and https://www.analyticscalculators.com/calculator.aspx?id=5 (accessed date 30 August 2021).

## 3. Results

The study groups differed in the prevalence of depressive symptoms measured by the BDI scale. The score indicating depression was most frequently reported in Group 1. Group 1 also had the highest number of cases indicating moderate to severe depression (Table 2).

The study groups did not differ in the prevalence of anxiety as a state and as a trait. The highest anxiety as a state was most frequently reported in Group 3 (Table 3).

In groups 1 and 3, high life satisfaction was found in about 60% of the subjects. In the pre-dialysis patients (Group 2), the result indicating a high life satisfaction was achieved only in about 40% of the subjects. This relationship demonstrated statistical significance (Table 4).

The analysis of questionnaires and tests revealed Group 1 to exhibit the lowest handgrip strength of both the right and left hand, which was significantly lower compared to Group 2. The lowest satisfaction with life (SWLS) was observed in Group 2. 

The SWLS scores obtained by group 2 were found to be significantly lower compared to group 1 and Group 3. The groups of respondents did not differ significantly in the total score obtained in the BDI scale. However, a separate analysis of the emotional and somatic part of the scale indicated a significantly higher value of the somatic part in group 1 compared to group 2. The level of anxiety examined using STAI X-1 and STAI X-2 questionnaires was similar in all groups and not significant (Table 5 and Table 6).

In each of the three groups, nearly half of the patients exhibited depressive symptoms (Table 2); therefore, the results of non-depressed (BDI < 12) and depressed (BDI ≥ 12) individuals were compared in each group, with the biggest differences observed in Group 1. Patients in this group with depressive symptoms had significantly lower handgrip strength, lower reported life satisfaction (SWLS) and higher anxiety as a state (STAI X-1) and higher anxiety as a trait (STAI X-2). Patients in group 2 (non-dialysis) and Group 3 (post-transplant) with depressive symptoms, rated their life satisfaction lower (SWLS) and reported higher anxiety as a state (STAI X-1) and higher levels of anxiety as a trait (STAI X-2) (Table 7).

Additionally, correlation analysis was performed between the BDI scores and handgrip strength, life satisfaction and anxiety levels. In Groups 1 and 3, a statistically significant negative correlation was found between the BDI and handgrip strength, SWLS and a positive correlation between the BDI and the STAI. In Group 2, correlation significance was shown only for SWLS and STAI (Table 8). 

There was no significant relationship in the three study groups, between the emotional state of the respondents and age, the number of comorbidities, months of dialysis (Group 1), and time after kidney transplantation (group 3). 

The results of the multivariate regression performed for all patients combined (All) revealed that the biggest effect on the level of depression lies with one’s belonging to a defined group of subjects. This is evidenced by the highest absolute value of the B coefficient. The negative nature of the relationship indicates that higher BDI values were recorded in dialysis patients (Group 1) compared to pre-dialysis (Group 2) and post-transplant patients (Group 3). Regression analysis performed separately for each of the groups showed the strongest association between depression and anxiety as a trait (STAI X-2). All regression models were considered highly fit as evidenced by high determination coefficient R^2^ and low standard error (SE) values (Table 9).

## 4. Discussion

Depressive disorders are found to be the most frequently observed mental disorders in patients with chronic renal failure [4]. They cause a lot of negative consequences for the individual in respect of family roles, work competence, sexual function, mobility and quality of life [22,23]. In the presented studies, nearly 38% of all respondents had symptoms of depression. A high percentage of depressive disorders among CKD patients was also noted by other authors [2,24,25,26]. 

Depressive disorders occurred in nearly half of the patients in each of the three groups. The highest percentage of people with symptoms of depression (48%) was reported among dialysis patients with stage V CKD (Group 1). This group also included the highest number of moderate and severe depression cases (28%). Similar results of moderate and severe depression (38.5%) were obtained by Shafi and Shafi [27], where the percentage of people with depression in the entire group of patients was 72.4%. Also, in the studies by Ćwiek et al. [5], Alencar et al. [28] and Mosleh et al. [9], dialysis patients had the highest percentage of depressive disorders. 

In the presented studies, the group of dialysis patients with stage V of CKD was characterized by significantly more somatic symptoms of depression, compared to pre-dialysis patients. This group also obtained the lowest handgrip strength of the right and left hands. These results are consistent with the reports of other authors who emphasize, that there is a relationship between the strength of handgrip and the occurrence of depressive symptoms, also in middle-aged and elderly people with various chronic diseases [10,11,12]. 

The significance of the relationship between these two parameters was also confirmed by correlation analysis of the BDI results with handgrip strength in the three studied groups. The more depressive symptoms observed, the smaller the handgrip strength of the right and left hand, especially in dialysis patients and transplant patients. Marques et al. emphasize that in clinical practices the evaluation of handgrip strength can simply be used as an indication for screening tests to evaluate not only physical but also mental health [12]. Anxiety as a trait was found to be the factor most significantly associated with depressive symptoms in each of the three patient groups.

The level of anxiety in all three groups was low and there were no significant differences between the groups. In the entire study group, a high level of anxiety as a state concerned nearly 24% of patients, and anxiety as a trait– 18% of the respondents. Molesh et al. [9] obtained similar results referring to the occurrence of high-level anxiety (19.7%). In studies by Shafi and Shafi [27], moderate to severe anxiety symptoms were present in 34.6% of patients. In contrast, in studies by Baloch and Almagir [29], as many as 50% of patients with stage V of CKD had moderate to severe anxiety symptoms. The regression analysis, however, showed that anxiety as a trait is a highly significant factor associated with depression in each of the three groups. This result is not surprising, as anxiety is an inherent component of depressive disorders, also affecting patients with CKD. However, it is worth noting that in these groups, it is the more anxious patients who are at the highest risk of experiencing depressed mood and depressive symptoms. 

A strong association of these factors was also confirmed by the results of analysis correlation carried out in separate groups of patients. In all three groups, a high level of depression was significantly associated with higher values of anxiety as a state and as a trait. A similar relationship was also noted in the research by Rajan and Subramanian [30] and Cohen et al. [31]. Anxiety disorders have been linked to depression, lower perceived quality of life, and poorer behavioral adherence. 

The three study groups were significantly different in the level of perceived life satisfaction (SWLS). Surprisingly, the lowest perceived life satisfaction and the least patients with high life satisfaction were related to pre-dialysis patients. This shows how serious and burdensome it is to deal with a chronic disease, such as CKD. The diagnosis of CKD and a growing awareness of the need for lifestyle changes adapted to diagnostic and therapeutic procedures, the perspective of dialysis, a need for a transplant, and even a high mortality rate, are factors that strongly affect satisfaction and quality of life. It is worth mentioning that the pre-dialysis group included the youngest of all patient groups with the lowest number of comorbidities. These significant differences were not demonstrated in studies by Ćwiek et al. The SWLS scores were very similar in both hemodialyzed and non-dialyzed patients with CKD [5]. 

In this study, the largest number of patients with high levels of life satisfaction was observed in dialysis patients and patients following kidney transplantation. Similar results were noted by Purnell et al. [32] and Montes et al. [33]. In turn, Zegarow et al. [34] emphasized that satisfaction with life was not higher in kidney transplant patients. The correlation analysis showed a significant relationship between the mood of respondents and their level of satisfaction with life in each of the three examined groups. The more depressive the symptoms in patients, the lower the satisfaction with life. 

A comparative analysis of patients with symptoms of depression and without depressive disorders showed, that the level of life satisfaction and anxiety are factors that significantly differentiate patients regardless of the stage of the disease and treatment. An additional important factor was the handgrip strength, but only in the case of dialysis patients with stage V of CKD. 

The obtained results indicate that screening tests and monitoring of the emotional state of patients with CKD are necessary, regardless of the disease stage and treatment; this includes patients after kidney transplantation. The occurrence of depressive and anxiety disorders in the examined groups of patients promotes a constant search for new support tools and preventive measures to limit the development of mental disorders and improve quality of life. The divergence of the results published prompts us to conduct further research on these particular patient groups, with the unification of methodology and applied research tools.

## 5. Conclusions

The highest percentage of patients with depressive symptoms was recorded in the group of dialysis patients with stage V of CKD. The fewest patients with high life satisfaction were reported in the pre-dialysis patients. There was a significant relationship between the mood of the respondents and the level of anxiety and satisfaction with life in all groups, while a significant relationship between the mood and the handgrip strength occurred only in dialysis and kidney transplantation patients. Anxiety as a trait was the factor most significantly associated with depressive symptoms in each of the three groups. Screen testing and monitoring of the emotional state of patients with CKD are needed, regardless of the stage of the disease and treatment, including patients following kidney transplantation.

### Limitations

The tests performed, particularly those relating to depressive and anxiety disorders were screening in nature and the results were not equivalent to making a diagnosis by a psychiatrist. The study did not take into account the functional status of patients and the duration of the disease which should be added in further studies.

## Figures and Tables

**Table 1:** Characteristics of study groups and significance of differences between groups for selected variables (the Kruskal–Wallis one-way ANOVA test).

		Group 1 (*n* = 87)		Group 2 (*n* = 94)		Group 3 (*n* = 102)			
		Median	IQR	Median	IQR	Median	IQR	*p*	Cohen’s d
Age		62.00	19.00	53.50	23.00	59.00	26.00	0.0046 *	0.36
Number of comorbidities **		4.00	2.00	3.00	2.00	4.00	3.00	0.0019 *	0.40
Body height [m]		1.69	0.16	1.70	0.12	1.70	0.13	N	0.07
Body mass [kg]		74.00	21.50	76.00	22.00	76.00	15.00	N	0.15
BMI		26.09	5.22	25.56	6.82	26.17	6.67	N	0.17
Dialysis vintage [months]		75.00	106.00	-	-	24.00	30.00	-	-
Time since transplant [months]		-	-	-	-	25.00	29.00	-	-
		***n***	**%**	***n***	**%**	***n***	**%**	***p***	**Cohen’s d**
Sex	women	42	48%	48	51%	40	39%	0.2185	0.21
	men	45	52%	46	49%	62	61%		
Education	primary	6	6%	0	0%	4	4%	0.4327	0.29
	lower secondary	17	19%	17	18%	24	24%		
	upper secondary	34	39%	33	35%	36	36%		
	tertiary	31	35%	44	47%	37	37%		
Place of residence	village	4	5%	13	14%	24	24%	0.0045 *	0.47
	town	27	31%	37	39%	34	34%		
	city	56	64%	45	47%	43	43%		
Marital status	married/partnership	11	12%	21	22%	26	25%	0.3674	0.25
	widower/widow	58	67%	53	56%	60	59%		
	single	18	21%	21	22%	16	16%		

IQR—inter-quartile range; N—not statistically significant; *p*-value of Kruskal–Wallis one-way analysis of variance; *p* value of χ^2^ test; BMI—Body Mass Index; * *p* < 0.05; ** comorbidities: diabetes, hypertension, coronary heart disease, peripheral arterial occlusive disease.

**Table 2:** Prevalence of depression in the three groups (the χ^2^ test).

*n* (%)	No Depression (BDI < 12)	Depression (BDI ≥ 12)	*p*	Cramer’s V	Mild Depression (BDI 12–19)	Moderate Depression (BDI 20–25)	Severe Depression(BDI 26–63)	*p*	Cramer’s V
Group 1 (*n* = 87)	46 (53%)	41 (47%)	0.0814	0.27	25 (29%)	10 (11%)	6 (7%)	0.0328 *	0.03
Group 2 (*n* = 94)	63 (67%)	31 (33%)			24 (26%)	7 (7%)	0 (0%)		
Group 3 (*n* = 102)	68 (67%)	34 (33%)			28 (27%)	3 (3%)	3 (3%)		

BDI—Back Depression Inventory; *p*-value of χ^2^ test; * *p* < 0.05.

**Table 3:** Prevalence of anxiety in the three groups (the χ^2^ test).

	STAI X-1				STAI X-2			
	Low < 44	High ≥ 44	*p*	Cramer’s V	Low < 46	High ≥ 46	*p*	Cramer’s V
Group 1 (*n* = 87)	68 (78%)	19 (22%)		0.04	75 (86%)	12 (14%)	0.4648	0.07
Group 2 (*n* = 94)	72 (76%)	22 (24%)	0.8386		75 (80%)	19 (20%)		
Group 3 (*n* = 102)	76 (75%)	26 (25%)			82 (80%)	20 (20%)		

STAI X-1—State-Trait Anxiety Inventory as a state; STAI X-2- State-Trait Anxiety Inventory as a trait; *p*-value of χ^2^ test;

**Table 4:** Prevalence of low and high satisfaction with life in the three groups (the χ^2^ test).

	Low SWLS (5–17 Points)	Average SWLS (18–23 Points)	High SWLS (24–35 Points)	*p*	Cramer’s V
Group 1 (*n* = 87)	8 (9%)	26 (30%)	53 (61%)	0.0477 *	0.13
Group 2 (*n* = 94)	14 (15%)	42 (45%)	38 (40%)		
Group 3 (*n* = 102)	11 (11%)	31 (30%)	60 (59%)		

SWLS—Satisfaction with Life Scale, *p*-value of χ^2^ test; * *p* < 0.05.

**Table 5:** Descriptive results of the questionnaires and tests.

	Group 1 (*n* = 87)		Group 2 (*n* = 94)		Group 3 (*n* = 102)	
	Median	IQR	Median	IQR	Median	IQR
Right handgrip strength [kg]	24.00	17.70	30.00	22.00	29.60	17.40
Left handgrip strength [kg]	21.70	15.30	27.45	18.80	25.80	18.80
SWLS	25.00	8.00	22.00	5.00	24.00	7.00
BDI total	10.00	12.00	10.00	8.00	9.50	10.00
Emotional part	5.00	6.00	4.00	5.00	3.00	5.00
Somatic part	6.00	6.00	5.00	4.00	6.00	5.00
STAI X-1	39.00	13.00	40.00	8.00	34.50	17.00
STAI X-2	38.00	12.00	39.00	10.00	36.00	15.00

BDI—Back Depression Inventory; STAI X-1, X-2—State-Trait Anxiety Inventory; SWLS—Satisfaction with Life Scale; IQR—interquartile range.

**Table 6:** Significance of differences in the results between groups (the Kruskal–Wallis one-way ANOVA test and the post hoc test between groups).

	*p*	Group			Cohen’s d
		1 vs. 2	1 vs. 3	2 vs. 3	
Right handgrip strength [kg]	0.0152 *	0.0130 *	N	N	0.31
Left handgrip strength [kg]	0.04750 *	0.0487 *	N	N	0.24
SWLS	0.0018 *	0.0026 *	N	0.0193 *	0.40
BDI total	N	N	N	N	0.24
Emotional part	N	N	N	N	0.19
Somatic part	0.0091 *	0.0075 *	N	N	0.33
STAI X-1	N	N	N	N	0.18
STAI X-2	N	N	N	N	0.19

BDI—Back Depression Inventory; STAI X-1, X-2—State-Trait Anxiety Inventory; SWLS—Satisfaction with Life Scale; *p*-value of Kruskal–Wallis one-way analysis of variance and *p*-value of post hoc test between groups; * *p* < 0.05; N—not statistically significant.

**Table 7:** Within-group differences between subjects with and without depressive symptoms (the Mann–Whitney test).

		No Depression		Depression			
Group		Median	IQR	Median	IQR	*p*	Cohen’s d
Group 1	Right handgrip strength [kg]	30.50	19.00	20.00	10.20	0.0003 *	0.85
	Left handgrip strength [kg]	30.00	14.50	19.70	10.30	0.0001 *	0.96
	SWLS	28.00	5.00	22.00	6.00	0.0000 *	1.07
	STAI X-1	33.00	13.00	41.00	6.00	0.0000 *	1.35
	STAI X-2	31.50	11.00	41.00	7.00	0.0000 *	1.61
Group 2	Right handgrip strength [kg]	35.00	20.50	25.00	22.00	0.1410	0.31
	Left handgrip strength [kg]	29.30	18.00	25.00	18.30	0.2995	0.22
	SWLS	23.00	5.00	20.00	7.00	0.0023 *	0.66
	STAI X-1	38.00	9.00	42.00	9.00	0.0012 *	0.71
	STAI X-2	36.00	10.00	44.00	8.00	0.0000 *	1.25
Group 3	Right handgrip strength [kg]	30.40	17.50	25.30	16.00	0.0618	0.38
	Left handgrip strength [kg]	27.50	19.25	23.25	14.80	0.1268	0.31
	SWLS	25.50	6.50	21.50	6.00	0.0004 *	0.75
	STAI X-1	30.00	13.00	46.00	7.00	0.0000 *	1.60
	STAI X-2	31.50	11.00	45.50	7.00	0.0000 *	1.78

STAI X-1, X-2—State-Trait Anxiety Inventory; SWLS—Satisfaction with Life Scale; IQR—inter-quartile range; *p*-value of U Mann–Whitney Test; * *p* < 0.05.

**Table 8:** The correlation (r coefficient) between BDI and handgrip strength, SWLS, and STAI in three groups.

	r	Right Handgrip Strength [kg]	Left Handgrip Strength [kg]	SWLS	STAI X-1	STAI X-2
BDI	Group 1	−0.45 *	−0.45 *	−0.48 *	0.60 *	0.74 *
	Group 2	−0.17	−0.15	−0.30 *	0.49 *	0.64 *
	Group 3	−0.27 *	−0.22 *	−0.39 *	0.71 *	0.79 *

BDI—Back Depression Inventory; STAI X-1, X-2—State-Trait Anxiety Inventory; SWLS—Satisfaction with Life Scale; r—Spearman’s correlation coefficient; * *p* < 0.05.

**Table 9:** A multivariate regression model for all patients combined (All) and for each study group separately, indicating the association of depression with selected variables.

	AII		Group 1		Group 2		Group 3	
	co. B	±95% CI	co. B	±95% CI	co. B	±95% CI	co. B	±95% CI
Group	−0.75 *	−1.39—0.1	-	-	-	-	-	-
Sex	−0.02	−1.31–1.26	1.67	−0.53–3.87	−0.70	−3.28–1.88	−0.26	−2.35–1.84
Age	0.12 *	0.08–0.15	0.16 *	0.08–0.25	0.06	0–0.12	0.12 *	0.06–0.18
Dominant handgrip strength [kg]	−0.06	−0.12–0	−0.04	−0.16–0.07	−0.08	−0.19–0.03	0.00	−0.09–0.1
SWLS	−0.06	−0.18–0.06	−0.14	−0.39–0.1	−0.07	−0.32–0.18	−0.03	−0.2–0.13
STAI X-1	0.10 *	0.01–0.19	−0.01	−0.23–0.21	0.13	−0.01–0.28	0.14 *	0.01–0.26
STAI X-2	0.45 *	0.34–0.55	0.53 *	0.27–0.79	0.40 *	0.23–0.57	0.44 *	0.29–0.58
R^2^	0.62		0.67		0.49		0.70	
SE	4.41		4.58		4.49		3.91	
Effect size (f^2^)	1.63		2.03		0.96		2.33	
Normality of residuals (*p* value)	0.7666		0.4015		0.6530		0.4707	

co. B—slope coefficient; CI—confidential interval; STAI X-1, X-2—State-Trait Anxiety Inventory; SWLS—Satisfaction with Life Scale; R^2^—coefficient of determination; SE—standard error; Normality of residuals—*p*-value Shapiro–Wilk Test; All—regression model created for all patients combined; * *p* < 0.05.

## Data Availability

The data presented in this study are available on request from the corresponding author.
